# Evaluating antibiotics for use in medicine using a poloxamer biofilm model

**DOI:** 10.1186/1476-0711-6-2

**Published:** 2007-02-15

**Authors:** Abi L Clutterbuck, Christine A Cochrane, Jayne Dolman, Steven L Percival

**Affiliations:** 1 University of Wales, Institute of Rural Studies, Aberystwyth, Ceredigion, Wales, SY23 3AL, UK; 2 University of Liverpool, Department of Veterinary Clinical Science, Division of Equine Studies, Leahurst, Neston, South Wirral, CH64 7TE, UK; 3 ConvaTec Wound Therapeutics™, GDC, First Avenue, Deeside Industrial Park, Deeside, CH5 2NU, UK

## Abstract

**Background:**

Wound infections, due to biofilms, are a constant problem because of their recalcitrant nature towards antibiotics. Appropriate antibiotic selection for the treatment of these biofilm infections is important. The traditional in vitro disc diffusion method for antibiotic selection uses bacterial cultures grown on agar plates. However, the form of bacterial growth on agar is not representative of how bacteria grow in wounds and other tissue sites as here bacteria grow naturally in a biofilm. The aim of this research was to test a more appropriate method for testing antimicrobial efficacy on biofilms and compare with the standard methods used for antibiotic sensitivity testing.

**Methods:**

Outer Membrane Protein analysis was performed on E.coli, Staphylococcus aureus, Pseudomonas aeruginosa, Proteus mirabilis and Acinetobacter juni when grown on Mueller Hinton agar ('quasi-biofilm state') and 30% Poloxamer hydrogel ('true- biofilm state). Susceptibility to antibiotics on 28 clinical isolates was determined using the modified Kirby Bauer disc diffusion method, on agar and 30% Poloxamer.

**Results:**

Similar outer membrane proteins [OMPs] were identified in bacteria grown in a biofilm state and on a 30% poloxamer hydrogel, which were very different to the OMPs identified in bacteria grown on Mueller-Hinton agar and broth. There was a significant difference between the means of the clearance zones around the antibiotic discs on standard agar and poloxamer gels [P < 0.05]. The zones of clearance were generally smaller for poloxamer-grown bacteria than those grown on standard agar. Diffusion distances of various antibiotics through agar and 30% poloxamer showed no significant difference [P > 0.05].

**Conclusion:**

The findings of this experiment suggest that poloxamer gel could be used as an appropriate medium on which to conduct biofilm antibiotic susceptibility tests as it enables bacteria to be grown in a state representative of the infected surface from which the culture was taken.

## Background

In natural environments, bacteria frequently grow in structured communities called biofilms. Biofilms are defined as bacterial populations adherent to each other and/or surfaces encased within a three dimensional matrix of extracellular polymeric substances [EPS] [1]. Biofilms can constitute a major problem to human health with many clinicians citing them as the cause of a variety of chronic bacterial infections [2]. Bacterial cells are protected by growing in a biofilm and although antibodies produced in response to biofilm antigens may eliminate the planktonic cells shed from the biofilm, they cannot reach the sessile cells within the biofilm and may damage surrounding tissue instead [3]. Similarly, antibiotic therapy often fails to eradicate biofilms, suppressing only the symptoms of infection by killing the planktonic cells [4]. Consequently, infections in animals and humans may persist for years with recurring symptoms after each period of antibiotic treatment until the colonised surface is surgically removed.

Whether in humans or animals, the antibiotic resistance of biofilms has a significant impact on health including increased morbidity and mortality [5]. The prolonged treatment of diseases and infections causes increased health costs and serious implications for both human and animal welfare. Currently, antibiotic selection is based on an antibiotic sensitivity test using the Kirby-Bauer disc diffusion method, developed in 1966 by Bauer and others [6]. Other methods have since been developed but the disc diffusion technique was adopted by the National Committee for Clinical Laboratory Standards [NCCLS] in 1975 and is still used today as the basis for disc diffusion standards [7].

Although the disc diffusion method of antimicrobial sensitivity testing has been described as a reliable, easy and inexpensive method of evaluating antimicrobial efficacy [8], recent research has indicated that the results from the disc diffusion test are open to interpretive error and that it is only useful as a preliminary screen for susceptibility testing [9]. Costerton et al. [3] stated that culturing bacteria for use in the susceptibility test transforms a biofilm forming pathogen into a planktonic lab-adapted strain. Thus, the problem with the standard antibiotic susceptibility test is that bacterial growth on agar is not representative of how bacteria grow naturally in tissue sites. Consequently, the current method of antibiotic selection assesses bacterial sensitivity in an unrealistic state.

In this present study poloxamer F127, a di-block copolymer of polyoxyethylene and polyoxypropylene, was used as a medium on which bacteria could be grown as a biofilm phenotype and express the characteristics more appropriate to the 'real world'. An initial experiment was undertaken to determine the molecular weight of the outer membrane proteins of *P. aeruginosa *grown on standard agar, poloxamer gel and in a biofilm on a microtitre plate to confirm whether bacteria express a biofilm phenotype on poloxamer as was found by Gilbert et al. [10]. The second experiment then involved antibiotic sensitivity testing on standard agar and poloxamer gel to compare results for a range of bacterial species.

In this present study two approaches were used to study the effectiveness of antimicrobial dressings on microorganisms. Firstly a wide range of aerobic bacteria and yeasts were tested using a standard agar assay [Kirby Bauer disc diffusion method [6] and a second method used a poloxamer technique to encourage the same strains of microorganisms to exhibit a more clinically relevant biofilm phenotype. Gilbert and others determined that *P. aeruginosa *cells grown on poloxamer hydrogel ('true' biofilm form) express outer membrane proteins between 78 and 87 kDa, which are not evident in cells grown on standard nutrient agar ('planktonic/quasi-sessile state') [10]. Consequently poloxamer gel cultures mimic many of the properties of biofilm-grown *Pseudomonas aeruginosa *[10]. This indicates that there is a phenotypic difference between *P. aeruginosa *cells grown on poloxamer hydrogel and nutrient agar, with only poloxamer grown cells resembling biofilm cells. It was found from Wirtanen's study [11] that bacteria which are grown in poloxamer have biofilm properties and associated enhanced biocide resistance [11]. Gilbert and colleagues suggested that bacteria grown in poloxamer hydrogels could be exposed to biocides to provide a reproducible method for testing the antimicrobial efficacy of biocides against biofilm bacteria [10]. Evidence of biofilm growth in the poloxamer model was also confirmed using confocal laser microscopy [12]. Sincock and other found that using microscopy, bacteria within poloxamer hydrogels grew to high densities, formed microcolonies and exhibited a biofilm phenotype. The poloxamer hydrogels have also been used to study biofilms of *Streptococcus mutans *in plaque [13], to look at homoserine lactones and biocide efficacy in biofilms [14] and also to study biofilms and coaggregation in the freshwater bacteria *Blastomonas natataria *and *Micrococcus luteus *[15].

In the current study we have utilised and adapted the science of Wirtanen's biofilm model [11] to provide a more clinically relevant method to test the effectiveness of antimicrobial dressings on biofilm microorganisms. The aim of this research was to test a more clinically relevant biofilm model for assessing the efficacy of antimicrobial agents against microorganisms of clinical and veterinary importance.

## Methods

### Source of bacterial isolates and identification

All isolates used in this study were isolated from routine clinical specimens submitted to the University of Liverpool Veterinary Teaching Hospital, Leahurst, Wirral, UK. All isolates were identified morphologically and biochemically by standard laboratory procedure.

### Outer membrane protein assay

#### Chemicals

Mueller-Hinton broth (MHB – Laboratory M, Bury, UK) and Mueller-Hinton agar (MHA – Laboratory M, Bury, UK) were used throughout. Poloxamer F127 was obtained from Univar (Essex, UK). All other chemicals and reagents were obtained from BDH (Poole, UK), Bio Rad (Hemel Hempstead, UK) or Sigma (Poole, UK).

#### Poloxamer hydrogels (biofilm phenotype induction)

Poloxamer F127 was incorporated into MHB at a concentration of up to 30% which was then refrigerated overnight (4°C). The dissolved poloxamer was then autoclaved and returned to the fridge. The liquefied poloxamer was then poured into Petri dishes in 20 ml volumes. Dishes were incubated overnight at 35°C before inoculation.

#### Biofilm cultures

Biofilm cultures of all bacteria were prepared by inoculating a 96 well microtitre plate (Nunclon^®^, Scientific Laboratory Supplies, Manchester, UK) with MHB containing a mid-log phase culture. A Nunc-TSP pin-lid (SLS, Manchester, UK) with 96 pegs was then placed onto the plastic microtitre plate so that the pins inserted into each well of the plate, which provided a surface for bacterial attachment. The wells, containing MHB, were inoculated with approximately 10^8 ^of the test bacteria (based upon McFarlane standards) and placed onto a rocker at 37°C. The pegs were colonized then for 24 h. After 24 hours the biofilm was determined by breaking several pegs from various points on the lid. The removed pegs were placed in microfuge tubes, washed in sterile saline (to remove planktonic cells) and biofilm cells were then harvested by sonicating in an ultrasonic water bath for 5 minutes at an amplitude of 50 Hz.

#### Preparation and analysis of cell envelopes

The preparation and analysis of cell envelopes were conducted according to the methods of Gilbert et al., [10]. In brief, cell suspensions harvested from MH broth cultures, poloxamer hydrogels and biofilm cultures were centrifuged at 10 000 g for 10 minutes at 15°C (Biofuge 13R, Heraus Sepatech, Fisher Scientific, Loughborough, UK). The resultant pellets were resuspended in 500 μl sterile physiological saline and placed in 1.5 ml Eppendorf tubes and sonicated in the water bath for 1 minute at 4°C. N-laurylsarcosine (10% w/v) was added to give a final concentration of 2% w/v. The samples were resonicated for 30 seconds and centrifuged (10 000 g, 1 hour) at 4°C. Pellets were resuspended in Laemmli sample buffer (Bio Rad, Hemel Hempstead, UK) and mercaptoethanol, 5% w/v, and heated for 5 minutes at 100°C. Sodium dodecylsulfate polyacrylamide gel electrophoresis (SDS-PAGE) was conducted with a 15% gel and molecular weight standards (2.5–200 kDa, Invitrogen, Paisley, UK), using sample volumes containing 10 μg protein. Gels were then stained with Coomassie Brilliant Blue G250 (BDH, Poole, UK) for 2 hours and then destained for 45 minutes. Molecular weights were analysed using the Gene Snap computer package (SynGene Bio-Imaging System, Cambridge, UK).

### Antimicrobial suceptibility test

#### Organisms

Twenty-eight bacterial organisms were evaluated in this study and included; *Acinetobacter *sp, *Actinobacillus equuli, Aeromonas hydrophilia, Bacillus *sp, *Bordetella bronchiseptica, Corynebacterium *sp., *Enterobacter cloacae, Enterococcus faecalis, Escherichia coli, Klebsiella sp, Listeria *sp, *Micrococcus sp, Morganella morganii, Nocardia asteroides, Proteus *sp, *Pseudomonas aeruginosa, Rhodococcus equi *and *Staphylococcus *sp. Also, three standard bacterial strains were used, namely: *Escherichia coli *NCIMB 12210, *Pseudomonas aeruginosa *NCIMB 12469 and *Staphylococcus aureus *NCIMB 12702.

#### Antibiotic suceptibility testing

Susceptibilities to various antibiotics were determined by modified Kirby-Bauer disk diffusion methods according to the Clinical Laboratory Standards Institute [16] on both agar and 30% Poloxamer hydrogels. In brief, colonies from an overnight culture of a bacterial isolate were suspended in sterile physiological saline until the density of the test suspension matched the turbidity standard which was the equivalent of a bacterial concentration of 3.0 × 10^8^/ml (McFarland Standard, BioMérieux, Marcy l'Étoile, France). MH agar and poloxamer gel plates were inoculated with 1 ml of bacterial suspension. The suspension was spread over the surface of the agar plates using a sterile 1 ml syringe and swilled around the surface of the poloxamer gel plates to ensure complete coverage. Plates were left for 5 minutes before excess fluid was removed using a sterile pipette. Sterile forceps were used to place the antimicrobial discs on the plates. The antimicrobial discs were then placed on both a MH agar and poloxamer gel plate, in duplicate for each bacteria. Plates were repeated in duplicate for each bacterial organism. Discs were evenly spaced approximately 15 mm from the edge of the plate. Each disc was gently pressed to ensure even contact with the surface of the medium. After overnight incubation at 35°C, plates were removed from the incubator. The diameter of the zone of clearance around each antimicrobial disc was measured with callipers, together with additional light enhancement, and recorded in millimetres. For discs with high efficacy for which the zone could not be measured, Non- Measurable (NM) was recorded. As the poloxamer gel formation is temperature dependent (liquid below 15°C), and readily reversible, whilst recording zones of inhibition the temperature of the Petri dishes were kept at constant at 25°C.

The following antibiotic impregnated discs were used: amoxicillin/clavulanic acid (30 μg), ampicillin/sulbactam (20 μg and 30 μg), ciprofloxacin (5 μg), clindamycin (10 μg), erythromycin (15 μg and 30 μg), imipenem (10 μg),; levofloxacin (5 μg), meropenem (10 μg), penicillin G (5 u); all from Oxoid (Oxoid Ltd; Basingstoke, Hampshire, England).

### Antibiotic diffusion investigation

This investigation was carried out in order to compare the diffusion rates of different antibiotics through MH agar and 30% Poloxamer gel (biofilm model). Different antibiotics with different molecular weights (MW) were chosen for this study. These included Ciprofloxacin (5 μg – MW 331.34), Doxycycline Hydrochloride (15 μg – MW 512.94), Gentamicin (15 μg – MW 653.21), Levofloxacin (5 μg – MW 361.37) and Meropenem (10 μg – MW 356.37). In separate experiments an antibiotic disk was placed in the centre of a Petri dish containing MHA or 30% poloxamer. Three 13 mm sterile filter paper disks (Whatman, UK) were placed next to the antibiotic disks in every Petri dish at various distances away from the antibiotic disk. The concept behind this is that over a 24 hour period the known antibiotic will diffuse through the agar or poloxamer gel and become impregnated into the filter disks placed at known distances from the central antibiotic disk. The newly impregnated filter disks was then be removed and their efficacy against a named organism, in this case *E.coli*, would be investigated using a zone of inhibition test (ZOI), according to NCCLS guidelines [16] on agar. Where a zone of clearing was detected around the newly impregnanted disc it would indicate that the antibiotic has diffused to that distance. This was repeated in triplicate.

## Results

### Outer membrane protein test

**Figure 1:**
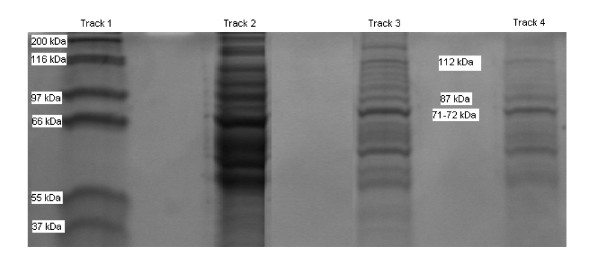
**SDS-PAGE gels of *Pseudomonas aeruginosa *after overnight incubation at 35°C**. Molecular weight standards are shown in track 1. The following tracks 2, 3 and 4 reveal the proteins from the planktonic culture grown on Mueller Hinton agar, the poloxamer hydrogels made from Mueller Hinton broth and the biofilm culture from the microtitre plate respectively.

Comparison of the outer membrane proteins of *P. aeruginosa *grown on poloxamer gel, Mueller-Hinton agar and the pin lid of the plastic microtitre plate (biofilm state) showed that the cells grown on poloxamer gel resembled the biofilm phenotype. The biofilm and poloxamer grown cells both expressed a protein at 87 kDa, a protein at 112 kDa and a protein at between 71–72 KDa which were not present in the MH agar grown cells (Figure 1). There were three proteins of similar weight around 57 kDa, 61 kDa and 64 kDa that were found in the *P. aeruginosa *cells from all three growth media. Also a 200 kDa protein was identified in the planktonic mode of growth and not in the biofilm grown bacteria.

For *Staphylococcus aureus *outer membrane proteins with weights of 103–104 kDa and 42–43 kDa were identified on 30% poloxomer. This corresponded to OMPs found from *Staphylococcus aureus *grown in the biofilm state but differed considerably from the OMPs identified in MH agar or MH broth.

The OMPs with weights of between 102 and 104 kDa and 19 kDa were identified from *Escherichia coli *grown on 30% poloxamer. These OMPs corresponded with OMPs found in *E.coli *growing in the biofilm state. As was the case with *Staphylococcus aureus *these OMPs differed considerably with the MH agar and MH broth grown bacteria.

For *Proteus mirabilis *OMPs at 289 and 205 kDa were identified when it was grown on 30% poloxamer. These were not evident in the planktonic state.

With *Acinetobacter juni *OMPs at 265 kDa, between 113 kDa and 115 kDa and between 60 and 61 kDa were identified on cells grown on 30% poloxamer. These OMPs corresponded to those grown in the biofilm state only and as above differed considerable from the planktonically grown cells.

### Antimicrobial resistance test

The results for the means of the zones of clearance around the antibiotics for the Gram positive and Gram negative bacteria on both MH agar and poloxamer gel are shown in tables 1 and 2. Five bacteria (*Corynebacterium pseudotuberculosis, Corynebacterium renale, Micrococcus *sp, *Staphylococus citreus *and *Staphylococcus hominis*) were excluded from analysis because the zones of clearance were measurable on poloxamer gel but were too big to be measured on agar with a number of antibiotics.

**Table 1:** Mean [± standard error] zones of clearance (mm) around all the antibiotics for Gram negative bacteria [NM – Not measured

	**ANTIBIOTIC**											
**BACTERIA**	**Test Method [mm]**	**Amoxycillin/Clavulanic acid**	**Ampicillin/Sulbactam**	**Ampicillin/Sulbactam**	**Ciprofloxacin**	**Clindamycin**	**Erythromycin**	**Erythromycin**	**Imipenem**	**Levofloxacin**	**Meropenem**	**Penicillin G**
		**30 **μg	**20 **μg	**30 **μg	**5 **μg	**10 **μg	**15 **μg	**30 **μg	**10 **μg	**5 **μg	**10 **μg	**5 **IU
*Acinetobacter sp*	Agar	12.80 ± 1.47	15.33 ± 0.67	15.40 ± 0.81	28.27 ± 0.37	9.53 ± 1.27	15.33 ± 0.75	25.27 ± 0.24	33.67 ± 2.03	27.33 ± 0.94	26.53 ± 0.33	8.93 ± 0.07
	Poloxamer	12.13 ± 0.18	13.07 ± 0.07	13.27 ± 0.18	17.00 ± 0.31	8.20 ± 0.00	9.73 ± 0.35	10.80 ± 0.00	18.80 ± 0.40	17.00 ± 0.00	18.27 ± 0.07	0.00 ± 0.00
*Actinobacillus equuli*	Agar	25.87 ± 0.59	28.27 ± 0.37	29.40 ± 0.60	26.73 ± 0.27	23.40 ± 0.20	20.93 ± 0.59	25.20 ± 0.20	35.47 ± 0.18	27.80 ± 0.20	31.33 ± 0.77	14.87 ± 0.35
	Poloxamer	22.40 ± 0.31	22.80 ± 0.40	23.67 ± 0.18	22.47 ± 0.24	11.27 ± 0.18	8.00 ± 0.12	11.33 ± 0.27	25.53 ± 0.18	23.13 ± 0.24	26.87 ± 0.13	18.20 ± 0.12
*Aeromonas hydrophilia*	Agar	14.07 ± 0.27	0.00 ± 0.00	7.40 ± 0.00	38.60 ± 0.20	7.53 ± 0.13	21.13 ± 0.13	24.07 ± 1.01	17.40 ± 0.12	36.40 ± 0.00	21.53 ± 0.07	7.20 ± 0.00
	Poloxamer	15.87 ± 0.24	7.73 ± 0.13	7.73 ± 0.18	27.80 ± 0.31	9.87 ± 0.13	15.53 ± 0.24	16.80 ± 0.20	16.47 ± 0.27	27.13 ± 0.07	18.00 ± 0.23	0.00 ± 0.00
*Bordetella bronchiseptica*	Agar	31.80 ± 0.20	17.73 ± 0.07	23.40 ± 0.31	32.00 ± 0.31	9.27 ± 0.27	23.93 ± 0.87	26.20 ± 0.23	33.60 ± 0.12	31.87 ± 0.07	46.47 ± 0.24	9.73 ± 0.18
	Poloxamer	22.67 ± 0.57	14.60 ± 0.20	16.67 ± 0.13	26.20 ± 0.53	8.67 ± 0.07	14.47 ± 0.29	15.87 ± 0.07	24.27 ± 0.55	25.67 ± 0.24	29.73 ± 0.07	0.00 ± 0.00
*Enterobacter cloacae*	Agar	10.00 ± 0.12	17.80 ± 0.00	19.27 ± 0.13	32.53 ± 0.29	16.33 ± 0.41	11.40 ± 0.35	11.67 ± 0.07	21.27 ± 0.13	27.20 ± 0.12	33.47 ± 0.71	7.40 ± 0.00
	Poloxamer	9.73 ± 0.18	11.00 ± 0.20	12.20 ± 0.12	21.73 ± 0.13	9.53 ± 0.18	9.40 ± 0.20	10.40 ± 0.12	18.53 ± 0.35	20.40 ± 0.23	22.33 ± 0.13	0.00 ± 0.00
*Escherichia coli 0117*	Agar	22.67 ± 0.94	23.40 ± 0.23	24.40 ± 0.42	35.13 ± 0.47	28.20 ± 0.23	27.40 ± 1.11	27.67 ± 0.13	28.87 ± 0.24	36.00 ± 0.42	33.60 ± 0.42	10.27 ± 0.44
	Poloxamer	14.93 ± 0.13	15.40 ± 0.31	15.80 ± 0.12	24.07 ± 0.07	9.20 ± 0.23	10.13 ± 0.18	11.87 ± 0.13	18.87 ± 0.29	23.47 ± 0.81	19.40 ± 0.35	0.73 ± 0.73
*Escherichia coli 08*	Agar	25.00 ± 0.00	22.27 ± 0.47	24.40 ± 1.60	37.93 ± 0.18	18.27 ± 0.27	18.93 ± 0.53	18.80 ± 0.53	30.13 ± 0.64	35.53 ± 0.37	35.47 ± 0.96	7.73 ± 0.35
	Poloxamer	15.20 ± 0.20	16.00 ± 0.12	16.33 ± 0.13	25.40 ± 0.40	7.80 ± 0.31	10.03 ± 0.54	11.80 ± 1.21	20.53 ± 0.18	25.27 ± 0.37	22.20 ± 0.40	0.00 ± 0.00
*Escherichia coli 0157*	Agar	24.13 ± 0.33	24.73 ± 0.74	26.13 ± 0.41	38.13 ± 0.07	8.80 ± 0.53	17.40 ± 0.31	15.00 ± 0.12	27.67 ± 0.13	34.53 ± 0.13	33.73 ± 0.18	8.07 ± 0.27
	Poloxamer	16.13 ± 0.13	16.33 ± 0.18	17.80 ± 0.20	24.20 ± 0.23	10.27 ± 0.24	10.33 ± 0.18	11.47 ± 0.07	21.07 ± 0.13	21.80 ± 0.12	21.80 ± 0.20	0.00 ± 0.00
*E.coli NCIMB12210*	Agar	21.80 ± 0.12	21.60 ± 0.00	22.20 ± 0.00	38.20 ± 0.12	9.80 ± 0.23	12.73 ± 0.07	13.60 ± 0.20	30.13 ± 0.07	36.27 ± 0.13	37.93 ± 0.18	NM
	Poloxamer	15.80 ± 0.12	16.47 ± 0.18	17.13 ± 0.24	26.40 ± 0.12	8.40 ± 0.00	11.60 ± 0.23	11.87 ± 0.07	19.93 ± 0.07	23.73 ± 0.27	20.60 ± 0.12	NM
*Klebsiella sp*	Agar	26.67 ± 0.35	21.33 ± 0.13	23.33 ± 0.41	34.27 ± 0.13	7.47 ± 0.18	11.87 ± 0.35	18.07 ± 0.07	28.40 ± 0.50	32.40 ± 0.31	31.07 ± 0.48	7.53 ± 0.33
	Poloxamer	14.60 ± 0.20	13.80 ± 0.12	14.87 ± 0.07	24.27 ± 0.27	7.20 ± 0.00	9.80 ± 0.42	11.93 ± 0.35	18.20 ± 0.31	23.13 ± 0.85	18.87 ± 0.18	0.00 ± 0.00
*Morganella morganii*	Agar	7.67 ± 0.07	15.33 ± 0.07	16.93 ± 0.13	25.20 ± 0.42	12.47 ± 0.18	8.07 ± 0.87	8.40 ± 0.60	19.00 ± 0.23	20.47 ± 0.18	30.33 ± 0.68	7.20 ± 0.00
	Poloxamer	9.00 ± 0.20	11.40 ± 0.31	12.60 ± 0.20	17.07 ± 0.44	7.27 ± 0.07	0.00 ± 0.00	7.20 ± 0.00	17.80 ± 0.12	16.53 ± 0.27	23.27 ± 0.18	0.00 ± 0.00
*Proteus vulgaris*	Agar	24.33 ± 0.33	21.47 ± 0.75	25.60 ± 0.70	41.27 ± 0.64	0.00 ± 0.00	0.00 ± 0.00	8.73 ± 0.64	22.80 ± 1.31	36.33 ± 0.70	12.53 ± 0.27	17.87 ± 1.10
	Poloxamer	17.33 ± 0.24	17.20 ± 0.60	19.00 ± 0.20	22.87 ± 1.95	11.07 ± 1.27	9.47 ± 0.33	11.47 ± 0.07	15.47 ± 0.35	24.27 ± 0.37	20.93 ± 0.24	13.47 ± 0.27
*Pseudomonas aeruginosa*	Agar	0.00 ± 0.00	0.00 ± 0.00	0.00 ± 0.00	36.47 ± 0.81	0.00 ± 0.00	13.73 ± 0.53	14.60 ± 1.03	22.60 ± 1.40	27.53 ± 0.87	33.00 ± 0.69	0.00 ± 0.00
	Poloxamer	0.00 ± 0.00	0.00 ± 0.00	0.00 ± 0.00	20.87 ± 0.07	0.00 ± 0.00	7.20 ± 0.00	7.80 ± 0.12	16.67 ± 0.35	17.03 ± 0.50	22.20 ± 0.20	0.00 ± 0.00
*P. aeruginosa NCIMB 12469*	Agar	NM	NM	NM	28.87 ± 0.07	NM	7.20 ± 0.00	7.40 ± 0.00	19.73 ± 0.41	21.60 ± 0.12	28.27 ± 0.24	NM
	Poloxamer	NM	NM	NM	20.73 ± 0.07	NM	6.87 ± 0.07	7.80 ± 0.12	17.40 ± 0.00	17.40 ± 0.00	22.33 ± 0.24	NM

**Table 2:** Mean [± standard error] zones of clearance (mm) around all the antibiotics for Gram positive bacteria [NM – Not measured

	**ANTIBIOTIC**											
**BACTERIA**	**Test Method [mm]**	**Amoxycillin/Clavulanic acid**	**Ampicillin/Sulbactam**	**Ampicillin/Sulbactam**	**Ciprofloxacin**	**Clindamycin**	**Erythromycin**	**Erythromycin**	**Imipenem**	**Levofloxacin**	**Meropenem**	**Penicillin G**
		**30 **μg	**20 **μg	**30 **μg	**5 **μg	**10 **μg	**15 **μg	**30 **μg	**10 **μg	**5 **μg	**10 **μg	**5 **IU
*Bacillus cereus*	Agar	13.87 ± 0.24	13.20 ± 0.00	14.93 ± 0.24	25.47 ± 0.07	25.73 ± 0.18	28.80 ± 0.23	30.07 ± 0.13	33.00 ± 0.64	24.87 ± 0.18	31.27 ± 0.68	7.07 ± 0.07
	Poloxamer	11.60 ± 0.00	11.80 ± 0.00	12.33 ± 0.13	17.13 ± 0.33	15.40 ± 0.23	16.73 ± 0.18	18.13 ± 0.07	22.40 ± 1.11	18.00 ± 0.12	22.33 ± 0.18	0.00 ± 0.00
*Bacillus licheniformis*	Agar	25.87 ± 0.24	23.47 ± 0.66	25.73 ± 0.13	34.67 ± 0.84	0.00 ± 0.00	30.80 ± 0.20	31.27 ± 0.55	39.27 ± 0.18	33.33 ± 0.18	41.53 ± 0.07	0.00 ± 0.00
	Poloxamer	18.13 ± 0.13	17.40 ± 0.00	18.20 ± 0.20	22.70 ± 0.32	8.73 ± 0.13	16.33 ± 0.07	17.53 ± 0.07	27.40 ± 0.12	21.60 ± 0.23	23.47 ± 0.18	9.40 ± 0.20
*Corynebacterium pseudotuberculosis*	Agar	NM	NM	NM	NM	NM	NM	NM	NM	NM	NM	NM
	Poloxamer	29.87 ± 0.13	27.33 ± 0.29	28.93 ± 0.47	31.53 ± 0.59	18.13 ± 0.18	25.87 ± 0.48	26.40 ± 0.64	35.33 ± 0.55	28.93 ± 1.23	30.13 ± 0.64	22.73 ± 0.29
*Corynebacterium renale*	Agar	NM	NM	NM	NM	NM	NM	NM	NM	NM	NM	NM
	Poloxamer	32.47 ± 0.75	31.20 ± 0.23	31.00 ± 0.69	20.20 ± 0.12	19.07 ± 0.35	22.33 ± 0.18	24.00 ± 0.00	35.53 ± 0.13	19.40 ± 0.00	30.47 ± 0.27	25.40 ± 0.31
*Enterococcus faecalis*	Agar	31.67 ± 0.13	27.07 ± 0.13	29.20 ± 0.00	20.87 ± 0.07	12.00 ± 0.20	24.53 ± 0.18	25.53 ± 0.24	29.67 ± 0.47	22.13 ± 0.24	23.27 ± 0.27	20.13 ± 0.64
	Poloxamer	16.80 ± 0.20	17.13 ± 0.24	18.60 ± 0.12	13.53 ± 0.18	8.20 ± 0.12	10.00 ± 0.20	10.47 ± 0.18	19.27 ± 0.18	13.13 ± 0.18	15.73 ± 0.13	12.27 ± 0.13
*Listeria ivanovii*	Agar	11.07 ± 0.27	7.20 ± 0.00	7.73 ± 0.07	36.07 ± 0.29	7.27 ± 0.07	9.87 ± 0.07	11.80 ± 0.83	36.60 ± 0.69	28.27 ± 0.47	25.40 ± 0.40	7.40 ± 0.12
	Poloxamer	8.67 ± 0.13	7.20 ± 0.00	7.20 ± 0.00	22.07 ± 0.41	7.20 ± 0.00	8.73 ± 0.07	8.67 ± 0.07	22.67 ± 0.07	20.73 ± 0.13	19.47 ± 0.41	0.00 ± 0.00
*Listeria monocytogenes*	Agar	22.87 ± 0.27	30.33 ± 0.75	32.60 ± 0.20	27.87 ± 0.24	24.87 ± 0.71	26.33 ± 1.98	28.00 ± 2.01	31.53 ± 0.85	26.00 ± 0.31	32.13 ± 0.97	25.07 ± 0.24
	Poloxamer	18.27 ± 0.66	16.67 ± 0.90	21.27 ± 0.37	13.07 ± 1.05	11.27 ± 0.77	15.60 ± 0.31	13.93 ± 0.13	20.13 ± 0.33	13.40 ± 0.31	13.00 ± 6.51	11.80 ± 1.11
*Micrococcus sp*	Agar	NM	NM	NM	NM	NM	NM	NM	NM	NM	NM	NM
	Poloxamer	34.40 ± 0.31	33.80 ± 0.20	34.33 ± 0.18	17.27 ± 0.68	22.80 ± 0.50	21.40 ± 0.20	22.80 ± 0.12	33.27 ± 0.77	16.60 ± 0.12	28.13 ± 0.13	24.33 ± 0.13
*Nocardia asteroides*	Agar	31.40 ± 0.90	14.03 ± 1.32	16.87 ± 0.70	37.33 ± 0.41	13.13 ± 0.33	14.27 ± 0.37	14.73 ± 0.13	35.13 ± 0.59	38.40 ± 0.61	21.87 ± 0.18	8.33 ± 0.44
	Poloxamer	23.60 ± 0.31	11.47 ± 0.07	12.40 ± 0.20	25.60 ± 0.12	13.33 ± 0.68	8.93 ± 0.13	10.33 ± 0.13	27.53 ± 0.18	25.07 ± 0.13	19.47 ± 0.07	0.00 ± 0.00
*Staphylococcus aureus*	Agar	23.73 ± 0.18	18.47 ± 0.55	20.73 ± 0.18	27.13 ± 0.18	30.40 ± 0.95	27.13 ± 1.49	27.07 ± 1.11	33.00 ± 0.64	27.13 ± 0.07	25.67 ± 0.24	14.00 ± 0.76
	Poloxamer	17.73 ± 0.07	13.60 ± 0.31	14.07 ± 0.13	18.60 ± 0.20	14.93 ± 0.37	15.13 ± 0.59	14.73 ± 0.18	23.73 ± 2.03	18.40 ± 0.53	18.80 ± 2.62	5.53 ± 2.78
*Staphylococcus aureus NCIMB 12702*	Agar	25.87 ± 0.07	24.80 ± 0.12	28.40 ± 0.12	22.60 ± 0.23	27.47 ± 0.27	21.87 ± 0.07	21.60 ± 0.12	32.27 ± 0.18	26.60 ± 0.20	36.47 ± 0.24	31.00 ± 0.00
	Poloxamer	22.33 ± 0.07	22.73 ± 0.07	17.07 ± 6.73	19.20 ± 0.00	16.53 ± 0.07	15.47 ± 0.24	15.53 ± 0.18	29.87 ± 0.07	19.60 ± 0.12	25.73 ± 0.07	20.27 ± 0.27
*Staphylococcus citreus*	Agar	NM	NM	NM	NM	NM	NM	NM	NM	NM	NM	NM
	Poloxamer	24.80 ± 0.00	23.80 ± 0.12	23.73 ± 0.79	17.80 ± 0.35	18.40 ± 0.12	19.93 ± 0.33	21.07 ± 0.07	30.80 ± 0.12	18.00 ± 0.12	26.93 ± 0.13	18.60 ± 0.00
*Staphylococcus epidermis*	Agar	33.07 ± 1.39	34.20 ± 0.53	35.27 ± 0.07	30.60 ± 0.70	30.80 ± 0.23	30.47 ± 1.17	34.00 ± 0.00	39.47 ± 0.27	30.60 ± 0.31	36.20 ± 0.92	33.67 ± 0.66
	Poloxamer	24.53 ± 0.13	24.93 ± 0.07	25.53 ± 0.07	19.93 ± 0.18	15.60 ± 0.83	15.47 ± 0.33	16.07 ± 0.18	28.53 ± 0.68	21.67 ± 0.35	25.13 ± 0.77	19.67 ± 0.35
*Staphylococcus hominis*	Agar	NM	NM	NM	NM	NM	NM	NM	NM	NM	NM	NNM
	Poloxamer	27.27 ± 0.29	26.87 ± 0.53	27.40 ± 0.20	16.33 ± 0.77	10.87 ± 0.33	18.53 ± 0.37	20.23 ± 0.15	30.87 ± 0.07	17.53 ± 0.13	26.80 ± 0.81	21.53 ± 0.47
*Staphylococcus hyicus*	Agar	34.47 ± 0.53	37.20 ± 0.12	38.60 ± 0.40	30.93 ± 0.13	28.33 ± 0.57	27.27 ± 0.07	28.60 ± 0.61	41.87 ± 0.13	29.00 ± 0.20	37.60 ± 0.00	38.00 ± 0.40
	Poloxamer	25.73 ± 0.07	25.80 ± 0.35	26.73 ± 0.24	21.67 ± 0.18	16.93 ± 0.07	14.40 ± 0.20	15.93 ± 0.07	31.60 ± 0.20	20.67 ± 0.13	27.00 ± 0.20	23.47 ± 0.13
*Staphylococcus intermedius*	Agar	33.00 ± 0.12	25.87 ± 0.07	25.33 ± 0.47	35.00 ± 0.40	28.00 ± 0.20	30.20 ± 0.42	27.40 ± 0.12	40.13 ± 0.41	32.20 ± 0.46	35.13 ± 0.41	9.13 ± 0.07

Amongst the 14 Gram negative bacterial species grown on MHA plates, ciprofloxacin was the most effective antibiotic, whereas, in the equivalent poloxamer gel grown organisms ciprofloxacin and meropenem were the most effective antibiotics. Of the 12 Gram positive bacteria tested, imipenem proved to be the most effective antibiotic against both the MH agar and poloxamer gel grown organisms, however it was the most effective in more of the organisms grown on poloxamer gel than those grown on MH agar (91.7% verses 58.3% respectively).

Although the same antibiotics were most effective in both the MH agar and poloxamer gel-grown Gram negative and Gram positive bacterial groups, antibiotic susceptibilities were often different between the two growth media. For example the bacterium *Nocardia asteroides *was most susceptible to levofloxacin when grown on MH agar, with an average 38.4 mm mean zone of clearance, however, when grown on poloxamer gel imipenem was the most effective antibiotic producing a 27.53 mm mean clearance zone.

As well as differences between the antibiotics for individual bacterial organisms, the efficacy of the same antibiotic also differed between the two growth media. This was most notable with the antibiotic penicillin G. Out of the 14 Gram negative organisms tested, penicillin G was the least effective antibiotic on both MH agar and poloxamer gel grown organisms (57.1 % and 64.3% respectively). However, only one of the organisms grown on MH agar displayed total resistance to penicillin G, in contrast to nine of the poloxamer grown organisms. Similarly, amongst the 12 Gram positive species, penicillin G was the least effective in 50% of organisms on both MH agar and poloxamer gel but whereas only one organism displayed resistance on MH agar, three organisms on poloxamer gel were completely unaffected by penicillin G. Therefore, whereas bacteria grown on MH agar often displayed some zone of clearance around penicillin G, poloxamer gel grown organisms often showed no clearance zone at all. For example, penicillin G was the least effective antibiotic against *Bacillus cereus *on both MH agar and poloxamer gel whereas the antibiotic was completely ineffective on the poloxamer gel grown culture, it produced an average 7.07 mm clearance zone on the MH agar plate.

*Aeromonas hydrophila *had larger zones of ampicillin-sulbactam and amoxicillin-clavulanic acid on poloxamer gels than MH agar. This was also true for *Bacillus licheniformis *when exposed to clindamycin. The significance of this result is under investigation.

### Antibiotic diffusion investigation

**Figure 2:**
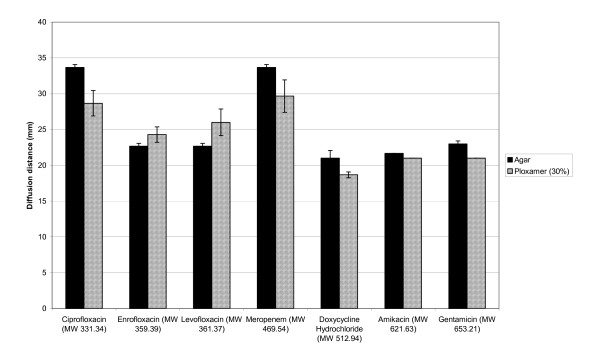
The average diffusion distances of various antibiotics through standard agar and 30% Poloxamer gels.

The average diffusion distances for each of the antibiotics through each of the two media, poloxamer gel and agar are shown in Figure 2. Clearly the diffusion rates through agar and 30% poloxamer were not significantly different (p < 0.05) for the antibiotics studied. In all cases the antibiotic had diffused a similar distance and shown to inhibit the growth of *E.coli *on agar plates.

## Discussion

The treatment of infections with topical or systemic antibiotics is becoming increasingly problematic due to the existence of biofilms [17,18]. Antibiotic sensitivity testing by traditional methods on agar are used to diagnose the best antibiotic to treat an infection. However, the choice and concentration of antibiotic are often unsuccessful at clearing the infection [19]. This is due to the fact that bacteria growing in a biofilm state are very recalcitrant to antibiotic treatment.

Gilbert et al. [10] suggested the use of poloxamer as a substitute for antimicrobial susceptibility testing and hypothesized that bacteria would grow in a biofilm state in poloxamer as opposed to a lab adapted 'planktonic' state. In their study OMPs from poloxamer grown and biofilm grown *Pseudomonas aeruginosa *had a number of identical OMPs which were not found in the bacteria when it was grown on agar and in broth (planktonic state). Within our study were have also discovered two outer membrane proteins in *Pseudomonas aeruginosa *at 87 kDa and 112 kDa in the biofilm and poloxamer grown state. These were not present in the MH agar grown cells which suggests that the protein profile of *Pseudomonoas aeruginosa *biofilm cells are different to that of MH agar grown 'planktonic/quasi-sessile' cells. The data generated in this paper supports the findings of Gilbert et al. [10], who found that poloxamer and biofilm grown *Pseudomonas aeruginosa *cells expressed outer membrane proteins between 78 and 87 kDa, which were not evident in MH agar grown cells. An additional protein between 71 and 72 kDa was found in the biofilm and poloxamer grown cells that was not found in the agar grown cells. This protein may represent the protein *OprC *[70 kDa] that was found in biofilm cells by Gilbert et al., [10]. This protein was not evident in the planktonic cells and imply that there is a phenotypic difference between *P. aeruginosa *cells grown on poloxamer gel and MH agar, with poloxamer gel grown cells resembling biofilm cells.

Overall for all the bacteria studied in this paper unique OMPs were identified when the bacteria were grown on poloxamer and in the biofilm state, that were not evident when the bacteria were grown on MH agar or in MH broth. OMPs were also identified from bacteria grown in MH agar and broth that were not found on poloxamer and bofilm grown bacteria. This suggests that bacterial cells display a biofilm phenotype in the presence of poloxamer. Consequently, this suggests that the sessile bacteria when grown on poloxamer express OMPs which are biofilm specific.

Having identified phenotypic similarities between poloxamer and biofilm grown cells an antimicrobial susceptibility test was conducted on a range of bacterial organisms grown in parallel on MH agar and poloxamer gel, in order to determine if a difference existed between the two different growth media. It was found that there was a significant difference [P < 0.05] between the growth diameters of the zones of inhibition on MH agar and poloxamer gel. The zones were generally smaller when the bacteria were grown on poloxamer gel and the antibiotic efficacy often differed between the two different media. For example, imipenem was the most efficient antibiotic against MH agar grown *Actinobacillus equuli *[35.4 mm mean diameter inhibition zone], whereas meropenem was the most effective antibiotic against the poloxamer gel grown form of the bacterium producing a 26.87 mm mean zone of inhibition. Not only was there a difference in the extent of antibiotic efficacy on both MH agar and poloxamer gel between antibiotics but the degree of efficacy also differed for the same antibiotic. Notably this was demonstrated in the case of penicillin G where organisms tested showed susceptibility when grown on MH agar but complete resistance when grown on poloxamer gel. It is also important to note that in this study the zones sizes are not comparable between the different antibiotics particularly as the methods employed are not quantitative, although gross differences can be concluded.

The differences in results relating to the two types of media calls into question the applicability of the traditional Kirby Bauer antibiotic susceptibility test which has been used widely in microbiology laboratories over the last forty years or so. Incorrect antibiotic concentrations can increase antibiotic resistance mutation rates in bacteria [20]. Generally, the use of ineffective antibiotics, whether due to class or dosage, to treat bacterial infections, will apply selection pressure to a population which will favour resistant strains. With the increasing threat of epidemic resistant organisms such as Methicillin Resistant *Staphylococcus aureus *(MRSA) the need for appropriate antibiotic selection is currently of prime importance to both clinical and veterinary science [21,22].

## Conclusion

Overall, this study has shown that the efficacy of antibiotics is reduced when bacteria are grown in the presence of poloxamer gel, as a biofilm phenotype. It has already been established that biofilm bacteria are resistant to antibiotics [23], however current susceptibility tests only use agar media that encourage bacteria to grow more within a 'planktonic/quasi-sessile' state than as a 'true' biofilm phenotype. The findings of this study suggest that poloxamer gel could be considered as an alternative medium on which to conduct antibiotic susceptibility tests as it enables bacteria to be grown in a biofilm state more representative of a biological surface infection (e.g. chronic infected wound). However, further studies are necessary to substantiate this claim particularly a quantitative version of this technology to aid clinicians and microbiologists to make informed decisions regarding prevention and treatment of serious biofilm infections.

## Competing interests

SLP and JD are employees of ConvaTec Wound Therapeutics™.

## Authors' contributions

ALC and JD performed experimental work. ALC, CC and SLP designed the study, collected and analysed the data and drafted the manuscript. All authors read and approved the final manuscript

## References

[B1] Costerton JW (1995). Overview of microbial biofilms. J Ind Microbiol.

[B2] Parsek MR, Fuqua C (2004). Biofilms 2003: Emerging themes and challenges in studies of surface- associated microbial life. J Bacteriol.

[B3] Costerton JW, Veeh R, Shirtliff M, Pasmore M, Post C, Ehrlich G (2003). The application of biofilm science to the study and control of chronic bacterial infections. J Clin Inves.

[B4] Stewart PS, Costerton JW (2001). Antibiotic resistance of bacteria in biofilms. Lancet.

[B5] Livermore DM (2003). Bacterial resistance: Origins, epidemiology, and impact. Clin Inf Dis.

[B6] Bauer AW, Kirby WMM, Sherris JC, Turck M (1966). Antibiotic susceptibility testing by a standardized single disc method. Am J Clin Pathol.

[B7] Wheat PF (2001). History and development of antimicrobial susceptibility testing methodology. J Antimicrob Chemother.

[B8] Gaudreau C, Gilbert H (1997). Comparison of disc diffusion and agar dilution methods for antibiotic susceptibility testing of *Campylobacter jejuni *subsp. *jejuni *and *Campylobacter coli*. J Antimicrob Chemother.

[B9] Manoharan A, Pai R, Shankar V, Thomas K, Lalitha MK (2003). Comparison of disc diffusion and E test methods with agar dilution for antimicrobial susceptibility testing of *Haemophilus influenzae*. Indian J Med Res.

[B10] Gilbert P, Jones MV, Allison DG, Heys S, Maira T, Wood P (1998). The use of poloxamer hydrogels for the assessment of biofilm susceptibility towards biocide treatments. J Appl Microbiol.

[B11] Wirtanen G, Salo S, Allison DG, Mattila-Sandholm T, Gilbert P (1998). Performance evaluation of disinfectant formulations using poloxamer-hydrogel biofilm-constructs. J Appl Microbiol.

[B12] Sincock SA, Rajwa B, Robinson PJ (6451). Characteristics and dynamics of bacterial populations with poloxamer hydrogel biofilm constructs. Abstract International Society for Analytical Cytology XX International Congress, May 20–25, 2000, Le Corum, Montpellier, France.

[B13] Kim MM, Park HK, Kim SN, Kim HD, Kim YH, Rang MJ, Ahn HJ, Hwang JK (3883). Effect of a new antibacterial agent, xanthorrhizol on the viability of plaque biofilm. Poster IADR/AADR/CADR 80th, San Diego, March 6–9th 2002.

[B14] MacLehose HG, Gilbert P, Allison DG (2004). Biofilms, homoserine lactones and biocide susceptibility. Journal of Antimicrobial Chemotherapy.

[B15] Rickard AH, Gilbert P, Handley PS (2004). Influence of growth environment on coaggregation between freshwater biofilm bacteria. J of Applied Microbiology.

[B16] National Committee for Clinical Laboratory Standards (2000). Methods for dilution antimicrobial susceptibility testing for bacteria that grow aerobically. Approved standard M7-A5 National Committee for Clinical Laboratory Standards, Wayne, Pa 5.

[B17] Percival SL, Bowler PG (2004). Biofilms and their potential role in wound healing. Wounds.

[B18] Percival SL, Rogers AA, McBain A, Allison D, Pratten J, Spratt D, Upton M, Verran J (2005). The significance and role of biofilms in chronic wounds. Biofilms: Persistence and ubiquity Biofilms: Persistence and Ubiquity, The Biofilm Club 7th Meeting of the Biofilm Club, Gregynog Hall, Powys 7–9th September 2005.

[B19] Ceri H, Olson ME, Stremick C, Read RR, Morck D, Buret A (1999). The Calgary biofilm device:new technology fro rapid determination of antibiotic susceptibilities of bacterial biofilms. J Clin Microbiol.

[B20] Martinez JL, Baquero F (2000). Mutation frequencies and antibiotic resistance. Antimicrob Agents Chemother.

[B21] Johnson AP, Aucken H, Cavendish S, Ganner M, Wale MCJ, Warner M, Livermore DM, Cookson BD (2001). Dominance of EMRSA-15 and -16 among MRSA causing nosocomial bacteremia in the UK: analysis of isolates from the European Antimicrobial Resistance Surveillance System [EARSS]. J Antimicrob Chemother.

[B22] Lloyd DH (1998). Chemotherapy; yesterday, today and tomorrow. Vet Dermatol.

[B23] Costerton JW, Stewart PS, Greenberg EP (1999). Bacterial biofilms: A common cause of bacterial infections. Science.

